# Early pathogenesis of Duchenne muscular dystrophy modelled in patient-derived human induced pluripotent stem cells

**DOI:** 10.1038/srep12831

**Published:** 2015-08-20

**Authors:** Emi Shoji, Hidetoshi Sakurai, Tokiko Nishino, Tatsutoshi Nakahata, Toshio Heike, Tomonari Awaya, Nobuharu Fujii, Yasuko Manabe, Masafumi Matsuo, Atsuko Sehara-Fujisawa

**Affiliations:** 1 Center for iPS Cell Research and Application (CiRA), Kyoto University, 53 Shogoin-Kawahara-cho, Sakyo-ku, Kyoto 606-8507, Japan; 2 Department of Growth Regulation, Institute for Frontier Medical Sciences, Kyoto University, 53 Shogoin-Kawahara-cho, Sakyo-ku, Kyoto 606-8507, Japan; 3 Department of Paediatrics, Kyoto University Graduate School of Medicine, Kyoto, Japan, 54 Shogoin-Kawahara-cho, Sakyo-ku, Kyoto 606-8507, Japan; 4 The Graduate School of Rehabilitation, Kobe Gakuin University, 518 Arise, Ikawadani-cho, Nishi-ku, Kobe 651-2180, Japan; 5 Department of Health Promotion Sciences, Graduate School of Human Health Sciences, Tokyo Metropolitan University, 1-1, Minamiosawa, Hachioji City, Tokyo 192-0397, Japan

## Abstract

Duchenne muscular dystrophy (DMD) is a progressive and fatal muscle degenerating disease caused by a dystrophin deficiency. Effective suppression of the primary pathology observed in DMD is critical for treatment. Patient-derived human induced pluripotent stem cells (hiPSCs) are a promising tool for drug discovery. Here, we report an *in vitro* evaluation system for a DMD therapy using hiPSCs that recapitulate the primary pathology and can be used for DMD drug screening. Skeletal myotubes generated from hiPSCs are intact, which allows them to be used to model the initial pathology of DMD *in vitro*. Induced control and DMD myotubes were morphologically and physiologically comparable. However, electric stimulation of these myotubes for *in vitro* contraction caused pronounced calcium ion (Ca^2+^) influx only in DMD myocytes. Restoration of dystrophin by the exon-skipping technique suppressed this Ca^2+^ overflow and reduced the secretion of creatine kinase (CK) in DMD myotubes. These results suggest that the early pathogenesis of DMD can be effectively modelled in skeletal myotubes induced from patient-derived iPSCs, thereby enabling the development and evaluation of novel drugs.

Duchenne muscular dystrophy (DMD) is characterised by progressive muscle atrophy and weakness that eventually leads to ambulatory and respiratory deficiency from early childhood^1^. It is an X-linked recessive inherited disease with a relatively high frequency of 1 in 3500 males^1^,^2^. *DMD*, which is responsible for DMD, encodes 79 exons and produces dystrophin, which is one of the largest known cytoskeletal structural proteins^3^. Most DMD patients have various types of deletions or mutations in *DMD* that create premature terminations, resulting in a loss of protein expression^4^. Several promising approaches could be used to treat this devastating disease, such as mutation-specific drug exon-skipping^5^,^6^, cell therapy^7^, and gene therapy^1^,^2^. Among these techniques, exon-skipping, which is a sequence-specific technique, has high efficacy and has potential for personalised medicine because of its specificity. However, it is still necessary to find drugs that are widely effective for many DMD patients despite the variability in their mutations.

The establishment of human induced pluripotent stem cells (hiPSCs)^8^ has led to a variety of new disease models^9^,^10^. Through their unlimited proliferation potential, patient-derived hiPSCs have advantages over patient-derived somatic cells such as myoblasts or fibroblasts in drug screening. Myoblasts from patients are the most common cell sources for assessing the disease phenotypes of DMD^11^,^12^. However, the degree of skeletal muscle damage in patients varies depending on factors such as genetic background, age, and medical history. Previous reports have shown that muscle cell differentiation from DMD patient myoblasts is delayed and that these cells have poor proliferation capacity compared to those of healthy individuals^11^,^12^. Furthermore, repetitive regeneration of DMD muscle leads to reduction in the proliferation potential of muscle satellite cells^13^,^14^. These phenotypes of DMD myoblasts are considered as secondary effects of chronic inflammation. In contrast, our study revealed that control and DMD myoblasts obtained by activating tetracycline-dependent MyoD transfected into iPS cells (iPS^tet-MyoD^ cells) have comparable growth and differentiation potential and can produce a large number of intact and homogeneous myotubes repeatedly. These properties permit the study of the early phenotypes of DMD that appear prior to inflammation.

The pathogenesis of DMD is initiated and progresses with muscle contraction. The degree of muscle cell damage at the early stage of DMD can be evaluated by measuring the leakage of creatine kinase (CK) into the extracellular space^15^. Several cell-damaging factors have been reported in DMD: accumulation of reactive oxygen species^16^, activation of nuclear factor kappa beta (NFκB)^17^, and calpain activity^18^. However, excess calcium ion (Ca^2+^) influx into skeletal muscle cells, together with increased susceptibility to plasma membrane injury, is regarded as the initial trigger of muscle damage in DMD^19^,^20^,^21^,^22^,^23^,^24^. Targeting these early pathogenic events is considered essential for developing therapeutics for DMD.

In this study, we established a novel evaluation system to analyse the cellular basis of early DMD pathogenesis by comparing DMD myotubes with the same clone but with truncated dystrophin-expressing DMD myotubes, using the exon-skipping technique. We demonstrated through *in vitro* contraction that excessive Ca^2+^ influx is one of the earliest events to occur in intact dystrophin-deficient muscle in response to electric stimuli. This event leads to extracellular leakage of CK in DMD myotubes. These results suggest that the early pathogenesis of DMD can be recapitulated with our system utilizing hiPSCs. Moreover, this system may enable the development of effective drugs that are applicable for most genetic variants of DMD by phenotypic screening based on early pathogenesis.

## Results

### Generation of tetracycline-inducible MyoD-transfected DMD patient-derived iPSCs (iPS^tet-MyoD^ cells)

**Figure 1:**
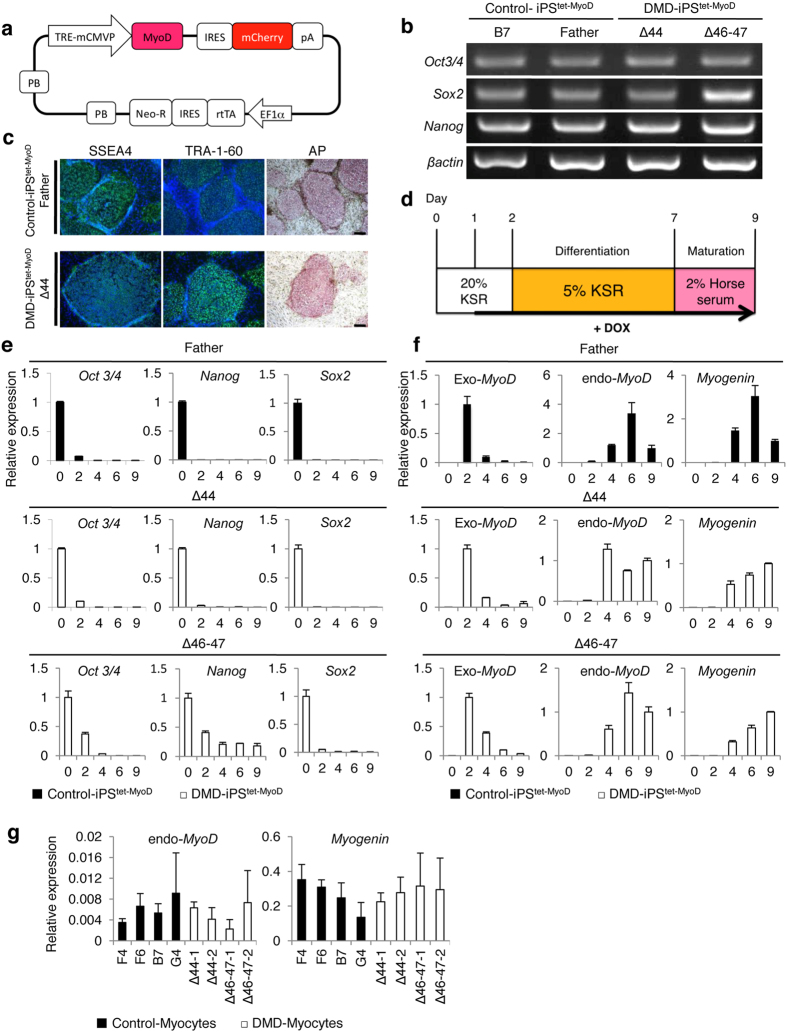
Generation and characterization of control and DMD patient-derived Tet-MyoD-transfected hiPS cells. (**a**) Construction of the tetracycline-inducible mCherry-linked *MyoD piggyBac* vector (Tet-MyoD). (**b**) RT-PCR analysis of Control-iPS^tet-MyoD^ (Father and B7) and DMD-iPS^tet-MyoD^ (Δ44 and Δ46–47) for pluripotency markers (*Oct3/4*, *Sox2*, and *Nanog*). (**c**) Immunocytochemistry for SSEA4 and TRA1–60 and alkaline phosphatase staining demonstrated the pluripotent state of Control-iPS^tet-MyoD^ Father and DMD-iPS^tet-MyoD^ Δ44. Scale bar, 200 μm. (**d**) Skeletal muscle induction scheme for Tet-MyoD-transfected hiPS cells. Differentiation was initiated with Dox addition at day 1. Cells were cultured with 20% knockout serum replacement (KSR) hiPS medium for the first 2 days. (**e**) Quantitative RT-PCR analysis of Control-iPS^tet-MyoD^ and DMD-iPS^tet-MyoD^ showing relative expression of pluripotency markers *Oct3/4*, *Nanog*, and *Sox2* and (**f**) myogenic markers exogenous- and endogenous-*MyoD* and *Myogenin* during the differentiation process. Data were normalised by setting day 0 = 1 for pluripotency markers, day 2 = 1 for exogenous-*MyoD*, and day 9 = 1 for endogenous-*MyoD* and *Myogenin*. n = 3. *Beta-actin* was used as an internal control. (**g**) Relative expression of endogenous-*MyoD* and *Myogenin* at day 9 of differentiation in each sample. Gene expression levels were normalised by setting levels in the human myoblast cell line Hu5/E18 at day 5 of differentiation = 1. *Ubiquitin C* was used as an internal control. Black and white bars indicate Control- and DMD-Myocytes, respectively.

Skin fibroblasts were biopsied from 2 different patients (age, 3 years 9 months and 8 years 11 months) who were diagnosed with DMD and had deletion of exon 44 (Δ44) and exon 46–47 (Δ46–47) in *DMD*, respectively, and from the 36-year-old biological father of the Δ44-patient. Human iPS cells were generated from these fibroblasts by a previously established method using retroviruses that carry the reprogramming factors *Oct3/4*, *Sox2*, *Klf4*, and *L-Myc*^8^,^25^. G4 and B7 were used as control cell lines^26^ and were both derived from the same person together with F4 and F6 from the father of Δ44 patient. *MyoD*, recognised as a master regulator gene for skeletal muscle differentiation^27^, was constructed as the tetracycline-inducible *MyoD piggyBac* vector (Tet-MyoD) (Fig. 1a). Subsequent transfection to control and DMD hiPS cells was performed using an established method that allows well-controlled myogenic differentiation to occur at high efficiency^26^. Introduction of the Tet-MyoD to DMD and control hiPS cells, hereafter referred to as DMD- and Control-iPS^tet-MyoD^, respectively, did not disrupt the pluripotent state^8^. These cells showed a flat and tightly packed cell morphology (Supplementary Figure S1a) and maintained the ability to form a teratoma (Supplementary Figure S1b). To verify the undifferentiated state of the established DMD- and Control-iPS^tet-MyoD^ clones, the expression of the pluripotent stem cell markers *Oct3/4*, *Sox2*, and *Nanog*^8^ in these clones was confirmed by reverse transcriptase-PCR (RT-PCR) (Fig. 1b). Expression of stage-specific embryonic antigen (SSEA)-4 and tumour-related antigen (TRA)-1–60, indicators for the undifferentiated stage of pluripotent stem cells, was detected by immunocytochemistry and alkaline phosphatase (AP) activity, further confirming that these hiPS cells retained stem cell characteristics after the introduction of the Tet-MyoD (Fig. 1c).

The skeletal muscle induction process consists of differentiation and maturation phases (Fig. 1d) and can be initiated by doxycycline (Dox) addition on day 1. The induction efficiency of MyoD was measured via flow cytometry using mCherry, and the efficiency of all clones was higher than 90%^26^ (Supplementary Figure S2a). Gene expression of *oct 3/4*, *nanog*, *sox 2*, exogenous- and endogenous-*MyoD*, and *myogenin* through differentiation was examined at days 0 (undifferentiated), 2, 4, 6, and 9. Upon activation of *MyoD* expression with Dox, pluripotent genes were significantly down-regulated (Fig. 1e), whereas genes for two endogenous myogenic regulatory factors, endogenous-*MyoD* and *Myogenin,* were up-regulated (Fig. 1f). Moreover, no endogenous or unrestrained expression of *MyoD* was detected from transfected Tet-MyoD at day 0 (Fig. 1f). The relative mRNA expression of endogenous-*MyoD* and *Myogenin* in each sample at day 9 of the induction demonstrated that differentiated skeletal muscle cells were comparable to skeletal muscle cells differentiated from the human myoblast cell line Hu5/E18^28^ (Fig. 1g).

### Morphologically and physiologically comparable intact myotubes differentiated from control and DMD-derived hiPSCs

**Figure 2:**
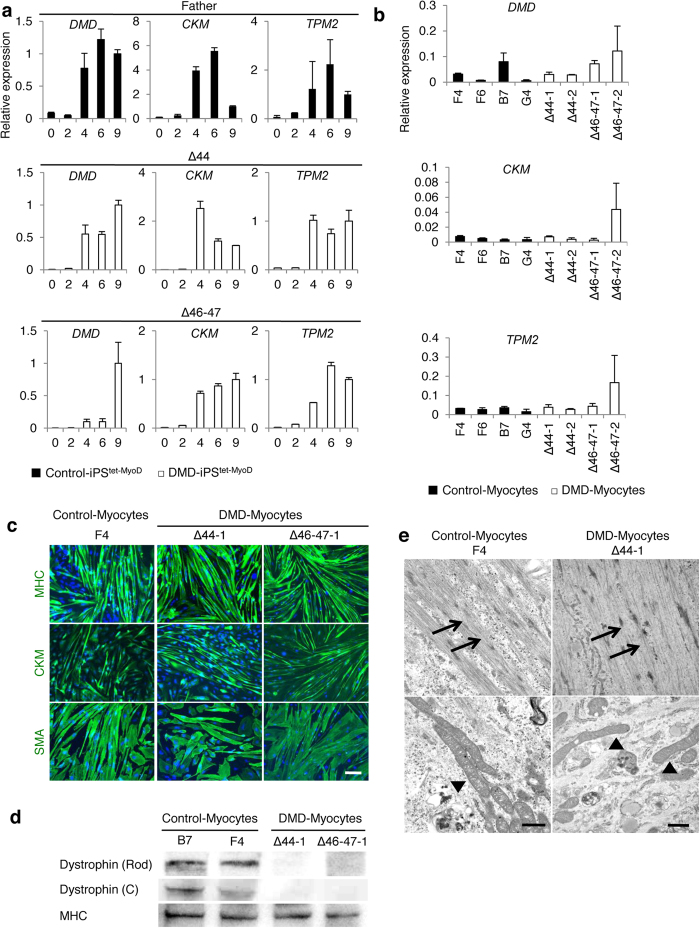
Morphologically and physiologically comparable skeletal muscle cells differentiated from Control-iPS^tet-MyoD^ and DMD-iPS^tet-MyoD^. (**a**) Quantitative RT-PCR of skeletal muscle marker expression in skeletal muscle cells induced from Control-iPS^tet-MyoD^ and DMD-iPS^tet-MyoD^, shown with black and white bars, respectively. Time course analyses were performed at days 0, 2, 4, 6, and 9. Relative mRNA levels of *DMD*, *CKM*, and *TPM2* were analysed. Data were normalised by setting data from day 9 = 1. n = 3. *Beta-actin* was used as an internal control. (**b**) Relative mRNA expression of *DMD*, *CKM*, and *TPM2* at day 9 in Control- and DMD-Myocytes. Gene expression levels were normalised to those of Hu5/E18 cells at day 5 of differentiation. *Ubiquitin C* was used as an internal control. (**c**) Immunocytochemistry of MHC, CKM, and skeletal muscle actin (SMA) in skeletal muscle cells induced from Control-iPS^tet-MyoD^ and DMD-iPS^tet-MyoD^ merged with DAPI. All immunofluorescence analyses were conducted at day 9 of differentiation. Scale bar, 200 μm. (**d**) Western blotting analyses of dystrophin protein expression in induced myotubes detecting the rod domain and C-terminus. The intensity of each band was normalised to that of myosin heavy chain (MHC). Cells were collected at days 9–12 of differentiation. (**e**) Electron microscopy images of induced skeletal muscle cells from Control-iPS^tet-MyoD^ and DMD-iPS^tet-MyoD^ at day 9. Myofibrils and mitochondria are indicated with arrows and arrowheads, respectively. Scale bars, 1 μm. 1:19,000 (Control) and 1:18,200 (DMD).

Transcripts of *DMD*, the skeletal muscle cell-specific gene *creatine kinase-muscle* (*CKM*), and *tropomyosin 2 (TPM2)*^29^, a Ca^2+^ channel marker recognised in matured contractible muscle cells, were up-regulated at day 4 subsequent to over-expression of *MyoD* in Control-iPS^tet-MyoD^ and DMD-iPS^tet-MyoD^ cell-derived myocytes (denoted as Control- and DMD-Myocytes, respectively) (Fig. 2a). Relative mRNA expression at the end of the differentiation process at day 9 indicated that the differentiated skeletal muscle cells from DMD-iPS^tet-MyoD^ and Control-Myocytes were comparable (Fig. 2b). In all iPSC-derived skeletal muscle cells, the expression levels of mature muscle differentiation markers were comparable to those of the human myoblast cell line Hu5/E18. Immunocytochemistry demonstrated that both Control- and DMD-Myocytes differentiated similarly to form myotubes that express myosin heavy chain (MHC), CKM, and skeletal muscle actin (SMA) at day 9 of induction (Fig. 2c). Differentiation efficiency was calculated based on the number of MHC-positive cells compared to the total number of nuclei. The clones showed comparable differentiation efficiency (Supplementary Figure S2b). Whereas the amount of transcripts of *DMD* in both Control- and DMD-Myocytes increased from day 4 (Fig. 2a), the dystrophin protein was not detected in DMD-Myocytes (Fig. 2d). Electron microscopy did not reveal any significant morphological differences between Control- and DMD-Myocytes (Fig. 2e). We also examined the functional properties of both Control- and DMD-Myocytes by applying electric stimulation to promote contractions^30^. As a result, stimulated contractions were observed (Supplementary Movie S1) in differentiated myotubes together with twitch-synchronised Ca^2+^ uptake using the fluorescent dye Fluo-8 (data not shown). These results indicate that DMD-iPS^tet-MyoD^ clones efficiently differentiated to form multi-nucleated myotubes physiologically and morphologically comparable to those of Control- Myocytes upon MyoD induction.

### Exon-skipping with AO88 restored expression of Dystrophin in DMD myotubes differentiated from DMD-iPS^tet-MyoD^ cells

**Figure 3:**
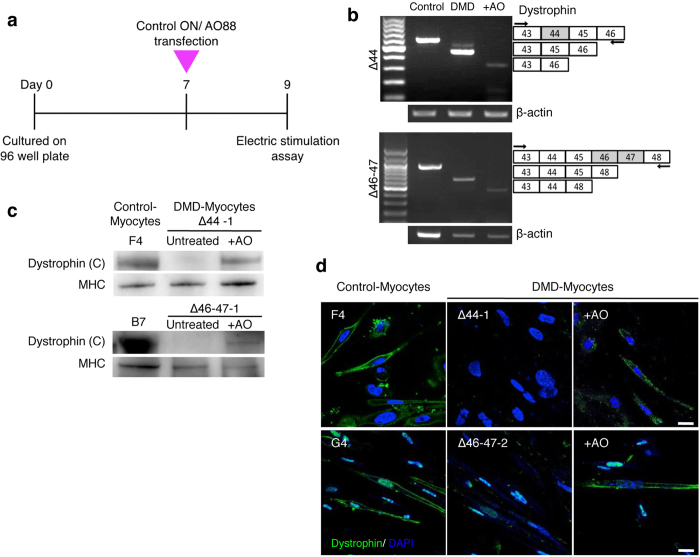
Restoration of dystrophin protein expression by AO88. (**a**) Transfection procedure for antisense oligonucleotide (AO) 88 in induced skeletal muscle cells. DMD-Myocytes were transfected with AO88 at day 7. Protein and RNA extraction was performed after 48 hours of transfection. (**b**) Exon 45 skipping was assessed by RT-PCR to compare Control-Myocytes (Father and B7) to DMD-Myocytes (Δ44 and Δ46–47). Exon 45 skipping led to an in-frame shift of exon 46 with Δ44 and exon 48 with Δ46–47, which resulted in smaller bands. (**c**) Western blotting analysis detecting the C-terminus of the dystrophin protein. AO-treated DMD-Myocytes showed restored expression of dystrophin protein. (**d**) Merged immunocytochemistry images for dystrophin and DAPI. Restored dystrophin protein expression was detected when samples were treated with AO88 (+AO). Scale bar, 20 μm.

To examine the phenotypic changes caused by the loss of dystrophin, we applied antisense molecules to DMD-Myocytes to induce dystrophin protein expression by altering the splicing pattern to lead an in-frame shift achieved by an antisense oligonucleotide (AO) complementary to the target site^31^. We introduced AO88, an RNA/ENA AO that is a fully phosphorothioate-modified form of AO85^32^; it specifically skipped exon 45 to promote translation by linking exon 43 to exon 46 in the Δ44-patient and exon 44 to exon 48 in the Δ46–47-patient (Fig. 3b). The most appropriate time to deliver AO88 is when *DMD* expression is assured (Fig. 3a), which is around day 7 of differentiation (Fig. 2a). Because the electrophoresis demonstrated shorter PCR products without any incomplete skipping remnants, exon-skipping denoted as+AO was conducted efficiently (Fig. 3b). Western blotting analysis indicated that exon 45 skipping generated dystrophin protein that has the C-terminal end (Fig. 3c). Immunocytochemistry confirmed that the dystrophin protein was localised peripherally along the cell membrane in Control-Myocytes and also in AO-applied DMD-Myocytes (+AO) (Fig. 3d). Similar to western blotting analysis, immunocytochemistry also demonstrated dystrophin protein expression in Control-Myocytes but not in DMD-Myocytes (Fig. 3d). From these results, we concluded that AO88 efficiently induced exon 45 skipping in DMD-Myocytes from two different patients to express a truncated dystrophin protein.

### Restored dystrophin expression attenuates Ca^2+^ overflow in DMD-Myocytes

**Figure 4:**
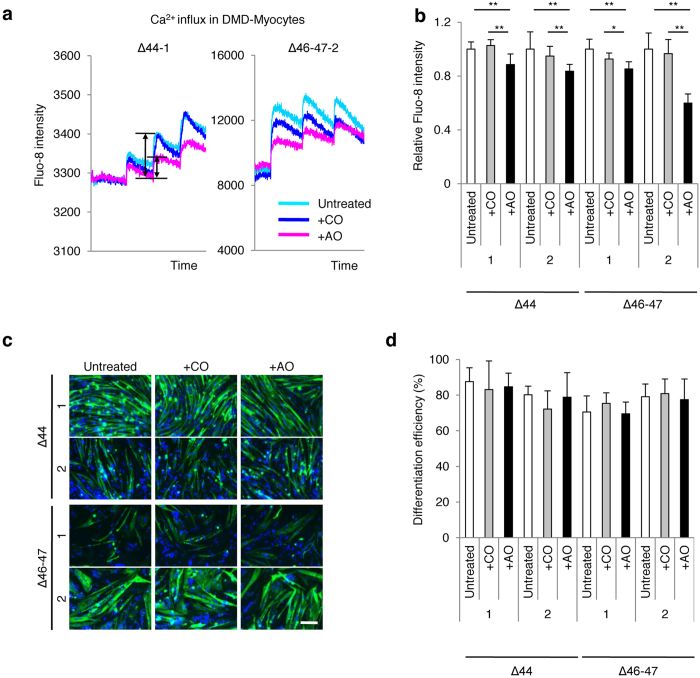
Restored expression of dystrophin diminishes Ca^2+^ influx in DMD muscle in response to electric stimulation. (**a**) Representative profiles of the Ca^2+^ influx pattern in Δ44 and Δ46–47 DMD-Myocytes determined through Fluo-8 intensity in response to electric stimulation. AO88 (+AO)- and control oligonucleotide (+CO)-treated DMD-Myocytes were compared with untreated DMD-Myocytes. Electric pulses were applied to the cells 5 sec after the stationary phase at a constant frequency of 0.2 Hz at 12 V for 1 min. The Ca^2+^ influx amplitude of AO-treated DMD-Myocytes was lower than that of untreated and + CO myocytes, as indicated by double arrowheads. (**b**) Quantitative analysis of Fluo-8 intensity amplification in Δ44 and Δ46–47 DMD-Myocytes. Amplification was normalised to that of untreated samples. n = 8, **p < 0.01. (**c**) Immunocytochemistry of MHC merged with DAPI in Δ44 and Δ46–467 DMD-Myocytes, compared with that of AO- and CO-treated DMD-Myocytes. Scale bar, 200 μm. (**d**) Quantitative analysis of the differentiation efficiency of two clones each from Δ44 and Δ46–47 DMD-iPS^tet-MyoD^. Percentages of MHC and DAPI double-positive fibres relative to total DAPI-positive cells at day 9 of differentiation. n = 4.

Myotubes were prepared on a 96-well plate and subjected to electric stimulation at 12 V, 0.2 Hz for 1 minute with Fluo-8. The fluorescence intensity, which indicates Ca^2+^ influx in myotubes, appeared to be synchronised with electrical excitement as assessed by an FDSS/μCell plate-reader system. Representative amplification graphs of Fluo-8 intensity illustrate higher Ca^2+^ influx rates in DMD-Myocytes compared to the control, as indicated by the double arrowheads (Supplementary Figure S3a). There was a significant difference in Ca^2+^ influx level between DMD and Control-Myocytes when the average amplification values of 3 different clones from each type of myotube were quantified (Supplementary Figure S3b). However, significant variance was seen in the Ca^2+^ influx for clones of the same myocyte type (Supplementary Figure S3c). Hence, for accurate measurements that eliminate the variability between clones, we compared Ca^2+^ influx within the same DMD-Myocytes clones by applying AO88. Ca^2+^ influx analysis was applied to 3 different types of DMD-Myocytes derived from the same patient: DMD-Myocytes (untreated), DMD-Myocytes treated with control oligonucleotide (DMD + CO), and DMD-Myocytes treated with AO88 (DMD + AO). Untreated DMD-Myocytes and DMD + CO exhibited significantly higher levels of Fluo-8 intensity than DMD + AO (Fig. 4a,b). Relative Fluo-8 intensities in the 2 different patients demonstrated a similar pattern, suggesting that the absence of dystrophin permits Ca^2+^ flow in excess compared to AO-treated DMD-Myocytes (Fig. 4b). To eliminate the possibility that these variances were resulted from differences in differentiation efficiency, we assessed the differentiation efficiency of each type of muscle cells. There were no significant morphological differences among untreated, DMD + CO, or DMD + AO treated skeletal muscle cells (Fig. 4c). Differentiation efficiency also did not show significant differences when all four clones were treated with CO or AO88 (Fig. 4d). Our synchronised electric stimulation system with Ca^2+^ visualisation and quantification supports previous reports indicating that excess Ca^2+^ flow occurs in DMD skeletal muscle cells^23^,^33^. Moreover, the truncated expression of dystrophin was sufficient to diminish increased Ca^2+^ flow in DMD myotubes. These findings suggest the necessity of the expression of the C-terminal end of the dystrophin protein to prevent excess Ca^2+^ flow in myocytes.

### Ca^2+^ influx provokes skeletal muscle cellular damage in DMD muscle

**Figure 5:**
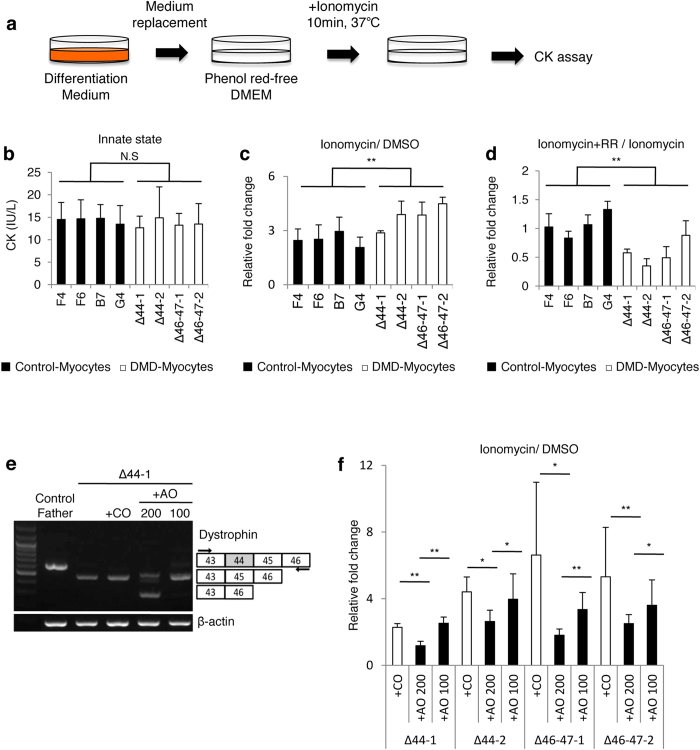
Ca^2+^ influx induces prominent skeletal muscle cellular damage in DMD-Myocytes. (**a**) Experimental design of the CK assay. (**b**) CK activity (IU/L) at the baseline condition of Control- and DMD-Myocytes, measured after 10 min of incubation at 37 °C. n = 6 with three independent experiments conducted for each assay. (**c**) Relative fold change in the CK value in Control- and DMD-Myocytes upon ionomycin addition, normalised against CK values for DMSO (control) addition. Two-way analysis of variance (ANOVA) revealed a significant difference in the rate of change between Control- and DMD- Myocytes in response to ionomycin treatment based on Scheffe’s test. **P < 0.01. n = 3 with three independent experiments conducted for each assay. (**d**) Relative fold change in the CK value with ionomycin and RR (a TRP family channel inhibitor) normalised to CK values with ionomycin treatment. Two-way ANOVA demonstrated a significant difference between control and DMD groups based on Scheffe’s test. **P < 0.01. (**e**) Exon 45 skipping with 2 different AO88 concentrations (100 nM and 200 nM) was assessed by RT-PCR and the results were compared to those for untreated and CO-treated DMD-Myocytes. Compared to 100 nM, 200 nM AO88 led to efficient exon 45 skipping, as demonstrated by a smaller band. (**f**) Relative fold change upon ionomycin addition in CO-treated and AO-treated DMD-Myocytes at 2 different concentrations was normalised to CK values in the case of DMSO (control) addition. n = 6, *P < 0.05, **P < 0.01.

To assess the effect of Ca^2+^ overflow on skeletal muscle cellular damage, cellular damage in the induced myocytes was evaluated by measuring the CK released in the supernatant (Fig. 5a). There were no significant differences in CK levels between Control- and DMD-Myocytes at baseline; the values ranged from 8 to 21 IU/L, with an average value of 14 IU/L (Fig. 5b). Previous reports have suggested that excessive influx of Ca^2+^ to myocytes indicates muscle cellular damage in muscular dystrophies^22^,^23^. Hence, to evaluate whether excess Ca^2+^ influx caused increased extracellular CK, ionomycin, an ionophore that induces Ca^2+^ influx in cells^34^, was added to measure CK activity. Addition of ionomycin indeed caused a dramatic change in CK activity: 2.9- to 4.5-fold in DMD-Myocytes and 2- to 3-fold in Control-Myocytes (Fig. 5c). Increased CK activity upon addition of ionomycin suggests that overflow of Ca^2+^ to the myotubes induces cellular damage. It has been reported that transient receptor potential (TRP) family channels are potential candidate molecules underlying DMD pathology. To determine whether the excess Ca^2+^ influx that occurred in DMD-Myocytes was attributed to the TRP family channel, ruthenium red (RR), a TRP family inhibitor^35^, was added to block Ca^2+^ channels. Addition of RR significantly reduced CK activity in DMD-Myocytes, but not in Control-Myocytes (Fig. 5d). We also confirmed that there were no changes in CK activity alone with RR treatment (Supplementary Figure S4a). These analyses suggest that TRP family Ca^2+^ channels partially mediate the excessive Ca^2+^ influx that provokes skeletal muscle cellular damage in DMD-Myocytes. To address the precise evaluation of exon-skipping efficiency, DMD-Myocytes were treated with two different concentrations of AO88 (100 nM and 200 nM), and the CK activity was measured. As shown by RT-PCR, the AO88 concentration affected the exon-skipping efficiency, as 100 nM AO88 treatment did not result in efficient exon 45 skipping, in contrast to 200 nM AO88 treatment (Fig. 5e). The effect of dystrophin protein expression on CK activity in DMD-Myocytes was also examined. The different exon-skipping efficiency led to differences in CK activity, where 200 nM AO-treated samples showed significantly decreased CK activity compared to CO- and 100 nM AO-treated DMD-Myocytes (Fig. 5f). To visualise Ca^2+^ behaviour, Fluo-8 was introduced with ionomycin. Induced Ca^2+^ overflow was observed in the cytoplasm of myocytes upon ionomycin addition (Supplementary Figure S4b). DMD-myocytes exhibited significantly higher Fluo-8 intensity in response to ionomycin addition than Control- or DMD + AO myocytes (Supplementary Figure S4c). Thus, the expression of truncated proteins prevented the cellular damage caused by Ca^2+^ overflow.

## Discussion

Experimental models of progressive diseases *in vitro* are indispensable for investigating primary phenotypes and pathogeneses, especially at the onset, and for the development of therapies and treatments. In this study, we have recapitulated the early pathogenesis of DMD by creating an *in vitro* DMD model with myotubes generated from patient-derived iPS cells.

Skeletal muscle differentiation in myoblasts from DMD patients is generally delayed compared to that in healthy individuals^11^,^36^,^37^. In addition, extracted myoblasts or myofibres from DMD patients are more likely to be exposed to inflammatory signalling molecules. Our differentiation system successfully induced the formation of myotubes from DMD patients, and the myotubes displayed analogous morphology and maturity compared with control myotubes, without any delay (Fig. 2a–c). One of the advantages of using hiPSCs is that they provide an intact and homogeneous skeletal muscle cell population with almost simultaneous myogenic differentiation in both Control- and DMD-iPS^tet-MyoD^ populations. Furthermore, our method eliminates the variability in genetic background and enables evaluation of phenotypic variation using an identical DMD clone. Comparing myotubes generated from patient-derived iPS cells with those derived from the same DMD clones but expressing dystrophin by application of the exon-skipping technique enabled us to demonstrate the primary cellular phenotypes in skeletal muscle solely resulting from the loss of the dystrophin protein (Fig. 4b). The exon-skipping technique, which alters the splicing pattern to an in-frame shift, has been employed in many DMD studies to convert the phenotype of cells to that of Becker muscular dystrophy (BMD)^1^,^6^,^38^. Although both DMD and BMD result from deletions or mutations in the same *DMD* gene, BMD patients have later onset and present milder symptoms^39^. Our results demonstrate that truncated but functional dystrophin protein expression improved the cellular phenotype of DMD myotubes. These findings suggest that the expression of functional dystrophin plays a critical role, particularly at the early stage of pathogenesis.

In DMD, the lack of dystrophin induces an excess influx of Ca^2+^ , leading to pathological dystrophic changes^22^. We consistently observed excess Ca^2+^ influx in DMD-Myocytes compared to Control-Myocytes (Supplementary Figure S3a and S3b) in response to electric stimulation. TRP channels, which are mechanical stimuli-activated Ca^2+^ channels^40^ that are expressed in skeletal muscle cells^41^, can account for this pathogenic Ca^2+^ influx. Previous reports have shown that suppression of TRP channels is accompanied by decreased Ca^2+^ influx in dystrophic fibres^21^. Moreover, it has been reported that Ca^2+^ influx induces muscular dystrophy in control myotubes through a TRP channel-dependent mechanism^20^ and that dominant-negative inhibition of Ca^2+^ influx via TRP channels ameliorates muscular dystrophy in animal models^19^. Thus, muscle fibres lacking dystrophin are more susceptible to unregulated Ca^2+^ influx, which leads to the activation of muscle degenerative pathways in DMD.

We have shown using identical clones that restoration of dystrophin protein expression via exon skipping in DMD-myotubes suppresses Ca^2+^ overflow compared to untreated DMD-Myocytes in response to electric pulses (Fig. 4a), resulting in the alleviation of cellular damage (Fig. 5c). Moreover, addition of RR to DMD-Myocytes suppressed the leakage of CK significantly (Fig. 5d). These results indicate that the TRP channel family contributes to the DMD pathology. Thus, our study reinforces previous findings and shows that excessive Ca^2+^ influx into intact myotubes under contraction is indeed one of the primary and cell-autonomous phenotypes of DMD. These results are the first to demonstrate the importance of the dystrophin protein in cellular damage prevention using identical DMD-Myocytes. Furthermore, these findings suggest that hiPSc-derived myocytes provide in-depth modelling of the primary DMD cellular pathology. Because we established a real-time Ca^2+^ imaging and CK activity assay system with uniformly differentiated myotubes in a 96-well plate, our systematic evaluation system may provide a practical and efficient method for future drug screening^42^. The present study also revealed that this experimental system can predict the effectiveness of exon skipping with customized antisense oligonucleotides in individual DMD patients by evaluating not only biochemical effects through western blotting (Fig. 3c) but also functional effects by measuring decreased Ca^2+^ influx into myotubes (Fig. 4a,b). Thus, our evaluation system is suitable for assessing the efficacy of exon-skipping drugs by phenotypic assay.

In conclusion, our study revealed that the absence of dystrophin protein induces skeletal muscle damage by allowing excess Ca^2+^ influx in DMD myotubes. Our experimental system recapitulated the early phase of DMD pathology as demonstrated by visualisation and quantification of Ca^2+^ influx using intact myotubes differentiated from hiPS cells. Thus, our iPS cell-based *in vitro* model of DMD is a valuable and powerful tool for investigating the mechanisms and pathogenesis of DMD. This evaluation system significantly expands prospective applications with regard to assessing the effectiveness of exon-skipping drugs in individual DMD patients and also enabling the discovery of general drugs that regulate the initial events in DMD such as Ca^2+^ influx by phenotypic screening.

## Methods

### Ethical approval

Approval for the study was received from the Ethics Committee of the Graduate School of Medicine, Kyoto University and the Kyoto University Hospital (approval number #824 and #G259) and conducted according to the guidelines of the Declaration of Helsinki. To protect confidentiality, all information about the patients is kept anonymous and written informed consent was obtained.

### Cell line and cell culture

Human dermal fibroblasts were donated from a 3-year, 9-month-old male and an 8-year, 11-month-old male, who have deletions in exon 44 and exon 46–47 of the *DMD*, respectively. Human dermal fibroblasts from the 36-year old biological father of Δ44-patient were also obtained as a control. All human iPS cells used in this study were established by retroviral overexpression of 4 transcription factors, *Oct3/4, Sox2, Klf4,* and *L-Myc,* as previously described^8^,^25^.

Four control hiPSCs, the father of Δ44-patient F4 and F6, G4 No. 35, and B7 No. 9^26^, and 2 DMD patient-derived iPS cells, Δ44 and Δ46–47, were cultured and maintained on inactivated mouse feeder cells, as previously described^8^, in primate ES cell medium (ReproCELL, Japan) supplemented with 4 ng/mL recombinant human basic fibroblast growth factor (bFGF, Wako).

### *MyoD* Transfection

The *MyoD* element was cloned into the PB-TAC-ERN vector using Gateway cloning to yield PB-h*MyoD* as previously described^26^.

hiPSCs were treated with 10 μM Y-27632 (Nacalai Tesque) at least 2 hours prior to the transfection experiment. Treated hiPSCs were dissociated into single cells with accutase (Sigma), and 1.0 × 10^6^ cells were resuspeneded in Opti-MEM (GIBCO). Plasmid DNA (5 μg of each), including PBase and PB-h*MyoD*, was transfected with an NEPA21 electroporator (Nepagene) using the conditions described in Table 1. Transfected cells were plated on mouse feeder cells at 2.0 × 10^5^ and 8.0 × 10^5^ cells/10 cm dish for clone selection and culture maintenance, respectively.

### Skeletal muscle induction

hiPS-MyoD cells were trypsinised into single cells and plated on Matrigel (BD Biosciences) or on a collagen I (Iwaki)-coated dish with 5.0 × 10^5^ to 1.0 × 10^6^ cells per well on 6-well plate dish in 20% KSR replacement media with 100 μg/mL neomycin sulphate (Nacalai Tesque) and 10 μM Y-27632 (Nacalai Tesque). The following day, culture medium was replaced with 1 μg/mL doxycycline (Dox; LKT Laboratories) in 20% KSR replacement media. Induction was carried out in 5% KSR/αMEM media with Dox and 2ME supplement for 5 days. At day 7 of induction, the medium was switched to 2% horse serum/DMEM media until day 14.

### Immunofluorescence analysis

Cultured cells were fixed with 100% methanol and subsequently blocked with Blocking One solution (Nacalai Tesque) at 4 °C. Fixed samples were layered in primary antibody, diluted in 10% Blocking One/0.01% Tween-20 (Santa Cruz)/PBS solution (PBST), and incubated at 4 °C for overnight. Following serial washes with PBST, cells were incubated with secondary antibody diluted in 10% Blocking One/PBST solution for 1 hour. DAPI, a nuclear stain (Sigma), was loaded at 1:5000 dilution for 5 min. Samples were observed and images were taken with a BZ9000 system (Keyence) and LSM 710 confocal microscope (Carl Zeiss) at 200 × and 400 × magnification, respectively. Fluo-8 (AAT Bioquest) intensities were assessed by the BZ9000 system. Antibodies used in this study are listed in Supplementary Table S1.

### Teratoma formation analysis

hiPSCs were suspended in Matrigel (BD, Biosciences) and inoculated into the tibialis anterior muscles of NOD/scid mice (Charles River Laboratories), which were previously treated with cardiotoxin under diethylether anaesthesia. Mice were dissected at 8–10 weeks after the cell transplantation.

Tumour sections were surgically removed and fixed in 4% paraformaldehyde (PFA)/PBS followed by substitution with 30% sucrose/PBS. Then, tumour samples were embedded in OCT compound (Tissue-Tek) and snap frozen on a carbon dioxide block. Frozen sections (10-μm thick) were prepared for haematoxylin and eosin (HE) staining and immunohistochemistry.

### Detecting Ca^2+^ influx via electric stimulation

Cells were differentiated on Matrigel-coated 96-well plates (Greiner). At day 9 of induction, cultured cells were loaded with Fluo-8, a fluorescent Ca^2+^ indicator (AAT Bioquest) and incubated at 37 °C for 30 min. The detection of Ca^2+^ through electric stimulation and the analyses were performed with an imaging plate reader FDSS/μCELL system (Hamamatsu photonics). Electric stimulation was applied with electrical field stimulation (EFS) of 96 multi-electrodes at 12 V with a 50 ms interval and a 0.2 Hz mono phase for 1 min after 5 s of resting phase. The sampling rate was set to 30 ms and fluorescence excitation was set at 480 nm with an LED excitation light source. Emission was set at 540 nm using an emission filter and detected with an electron multiplying CCD (EMCCD) camera. Measurements for 96 wells were performed under uniform conditions at 37 °C with simultaneous stimulation and detection.

### RNA extraction and quantitative real-time RT-PCR

Total RNA was extracted with Sepazol (Nacalai Tesque) following the manufacturer’s instructions. Extracted RNA was treated with DNase I (Invitrogen) to remove genomic DNA. cDNA synthesis was carried out with the Superscript III First-Strand Synthesis system for RT-PCR (Invitrogen) using either random hexamers or oligo dT as the primer. Quantitative real time RT-PCR was carried out with SYBR Green probe sets (Applied Biosystems) and a One Step thermal cycler (Applied Biosystems) and was performed in triplicate for each sample. *Beta-actin* and *ubiquitin C* were used as internal control for time course and day 9 differentiation analysis, respectively. Primer sets used in this study are listed in Supplementary Table S2.

### Protein extraction and Western blotting

Cells were lysed in 1% protease inhibitor cocktail (Roche) containing radio-immunoprecipitation assay (RIPA) buffer (50 mM Tris-HCl, pH 8.0, 150 mM NaCl, 1% Nonidet P-40 (NP-40), 1% sodium deoxycholate, and 0.1% SDS), by thorough sonication. Protein lysate (20–40 μg) was mixed with an equal volume of EzApply (ATTO) and boiled at 95 °C for 5 min. Samples were then separated on a 3–8% NuPAGE Novex tris-acetate mini gel (Invitrogen) at 150 V for 75 min with the addition of NuPAGE antioxidant (Invitrogen) during electrophoresis. The fractionated proteins were transferred to a nitrocellulose membrane using an iBlot system (Invitrogen) with the program, P0, 13 min. The membrane was blocked with Membrane Blocking Agent (GE Healthcare life sciences) and incubated with primary antibody at 4 °C overnight. After 3 washes with PBST, the membrane was incubated with secondary antibody for 1 hour at room temperature. Refer Supplementary Table S1 for detailed antibody information.

Detection was carried out with Pierce ECL western blotting substrate (Thermo scientific). Visualisation and semi-quantification of images were performed by a ChemiDox XRS + imaging system (BIO-RAD).

### Electron microscopy

Sample fixation and observation were carried out according to the Tokai Electron Microscopy, Inc. protocol that came with the instrumentation.

### Antisense oligonucleotide (AO) transfection

AO88 and control oligonucleotide (CO), which is a fully phosphorothioated RNA/ENA antisense oligonucleotide for a non-myogenic gene, were introduced at day 7 to induced skeletal muscle cells at the final concentration of 200 nM or 100 nM for 3 hours with Lipofectamine 2000 (Life Technologies) by the method previously described^43^. Cells were cultured in 2% horse serum/ DMEM medium for 48 hours and collected for protein and RNA extraction.

### CK value analysis

Cells were differentiated on Matrigel-coated 6-well plates. At day 9 of the induction, cells were washed with phenol red-free high glucose DMEM (Nacalai Tesque) and were replaced with the same media at 1 mL/well. Supernatant was collected from each well after 10 min of incubation at 37 °C and centrifuged at 1500 rpm for 5 min to eliminate any cell debris. Collected supernatants were supplemented with protease inhibitor cocktail (Nacalai Tesque) at 1:100 dilution and CK activity was measured by Oriental Yeast Co., LTD.

Ca^2+^ influx analysis was performed by adding 6.25 μM ionomycin (Wako) or ionomycin and 1 μM ruthenium red (Sigma) to cultured cells. Cells were then incubated for 10 min at 37 °C. Ca^2+^ uptake was visualised with Fluo-8 and measured with the BZ9000 system.

### Flow cytometry analysis of mCherry positive Cells

Doxycycline-treated cells were washed in Phosphate buffered saline (PBS) and incubated for 5 min with accutase to dissociate into single cells. After cells were counted, they were suspended in Hank’s balanced salt solution (Life technologies), supplemented with 1% BSA at 1.0 × 10^6^ cells/100 μL and analysed by an LSR Fortessa (BD Biosciences) for the expression of mCherry.

### Statistical analysis

All experiments were performed in triplicate and repeated at least twice. The data representing in all figures are means and standard deviation of the mean. Ca^2+^ uptake assays using electric stimulation were carried out with n = 12 for the Control- and DMD-Myocytes comparison and n = 8 for the DMD and DMD-Myocytes + AO comparison. The data in Figs 4b,d, 5e, S3b, S3c and S4c are presented using the two-tailed Student’s *t* test for independent samples by R software. The data in Fig. 5b–d, S2b and S4a are presented using the Scheffe’s test to compare Control-Myocytes with DMD-Myocytes samples by R software. Values of P < 0.05 were considered significant.

## Additional Information

**How to cite this article**: Shoji, E. *et al.* Early pathogenesis of Duchenne muscular dystrophy modelled in patient-derived human induced pluripotent stem cells. *Sci. Rep.* **5**, 12831; doi: 10.1038/srep12831 (2015).

## Supplementary Material

Supplementary Movie S1

Supplementary Information

## Figures and Tables

**Table 1:** Parameters for electroporation of Tet-MyoD piggyBac vector transfection in hiPSCs.

	Voltage(V)	Pulselength(msec)	Pulseinterval(msec)	No. ofpulses	Decayrate (%)	Polarity(+/−)
Poring Pulse	125.0	5.0	50.0	2.0	10.0	+
Transfer Pulse	20.0	50.0	50.0	5.0	40.0	+/−
