# Supplementary material for: Early pathogenesis of Duchenne muscular dystrophy modelled in patient-derived human induced pluripotent stem cells
Source: Sci Rep. 2015 Aug 20;5:12831. doi: 10.1038/srep12831 (PMC4642533; doi:10.1038/srep12831)
Supplement: Supplementary Information [file srep12831-s2.pdf]

Supporting information

Early pathogenesis of Duchenne muscular dystrophy modelled in patient-derived human induced pluripotent stem cells.

Emi Shoji[1,2], Hidetoshi Sakurai[1*], Tokiko Nishino[1], Tatsutoshi Nakahata[1], Toshio Heike[3], Tomonari Awaya[3], Nobuharu Fujii[5], Yasuko Manabe[5], Masafumi Matsuo[4], Atsuko Sehara-Fujisawa[2]

1. Center for iPS Cell Research and Application (CiRA), Kyoto University, 53 Shogoin-Kawahara- cho, Sakyo-ku, Kyoto 606-8507, Japan
2. Department of Growth Regulation, Institute for Frontier Medical Sciences, Kyoto University, 53 Shogoin-Kawahara-cho, Sakyo-ku, Kyoto 606-8507, Japan
3. Department of Paediatrics, Kyoto University Graduate School of Medicine, Kyoto, Japan, 54 Shogoin-Kawahara-cho, Sakyo-ku, Kyoto 606-8507, Japan
4. The Graduate School of Rehabilitation, Kobe Gakuin University, 518 Arise, Ikawadani-cho, Nishi-ku, Kobe 651-2180, Japan
5. Department of Health Promotion Sciences, Graduate School of Human Health Sciences, Tokyo Metropolitan University, 1-1, Minamiosawa, Hachioji City, Tokyo 192-0397, Japan

*Corresponding author: Hidetoshi Sakurai
Clinical Application Department, Center for iPS Cell Research and Application, Kyoto University, 53 Kawahara-cho, shogoin, Sakyo-ku, Kyoto, 606-8507, Japan.
Tel: +81-075-366-7055; Fax: +81-075-366-7074; Email: hsakurai@cira.kyoto-u.ac.jp

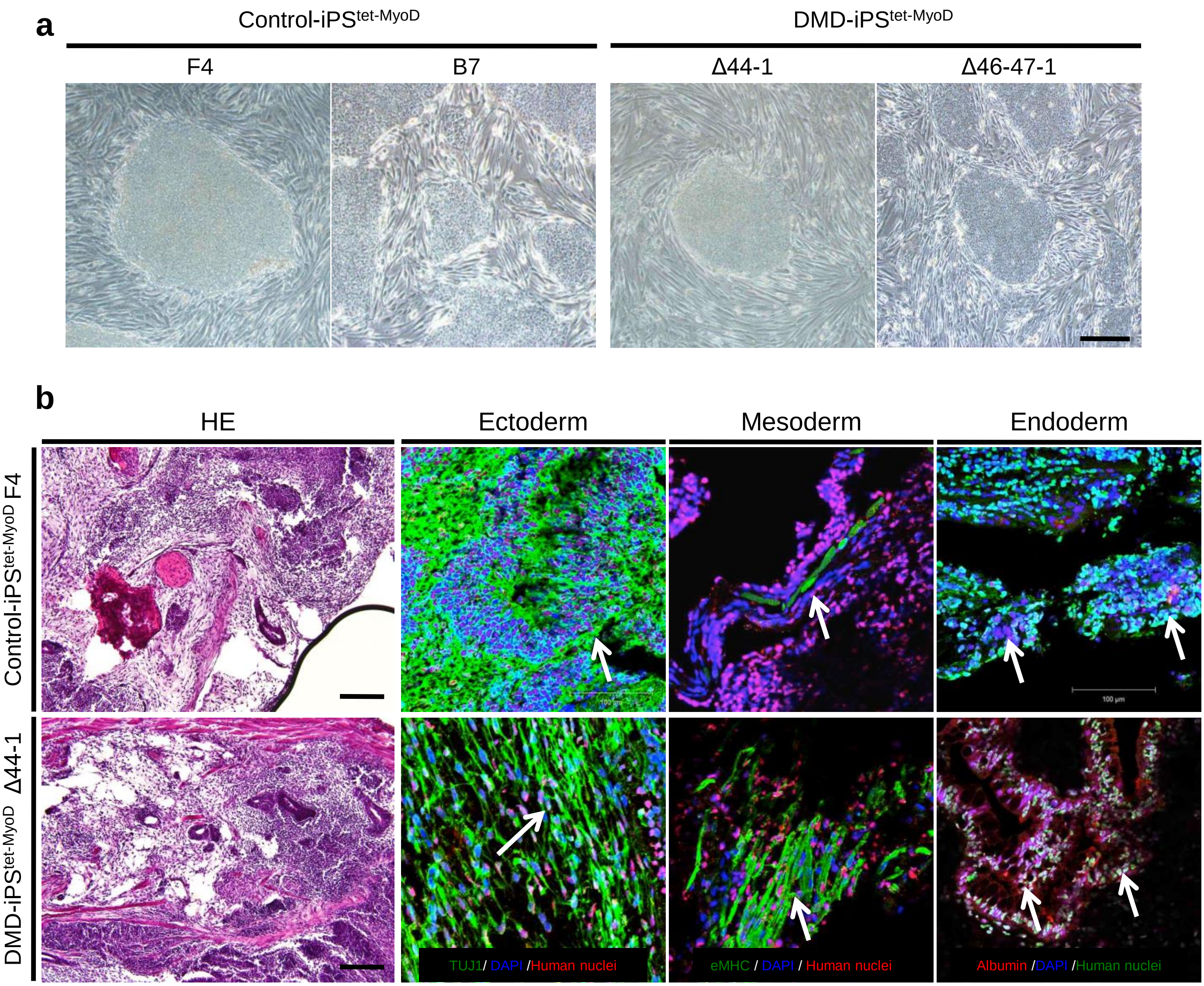

Supplementary Figure S1

(a) Morphology of Tet-MyoD transfected hiPS cells derived from 2 different DMD patients, who have deletion in exon 44 (DMDΔ44) and exon 46-47 (DMDΔ46-47) of the *DMD*, and from biological father of DMDΔ44 (Control, Father) and 201B7 (Control, B7). Scale bar, 200 µm. (b) Teratoma formation assay with hematoxylin and eosin staining (HE) and immunohistochemistry, showing ectoderm, mesoderm, and endoderm. Neuronal cells, skeletal muscle cells, and hepatocytes, were detected with Neuron-specific class III beta-tubulin (TUJ1), embyronic myocin heavy chain (eMHC), and albumin, respectively. Tumour sections were prepared from hiPSCs inoculated tibialis anterior (TA) muscles of NOD/scid mice. Arrows indicate endoderm, mesoderm, and ectoderm formed in TA muscles from each hiPSc clones. Scale bar, 100 µm.

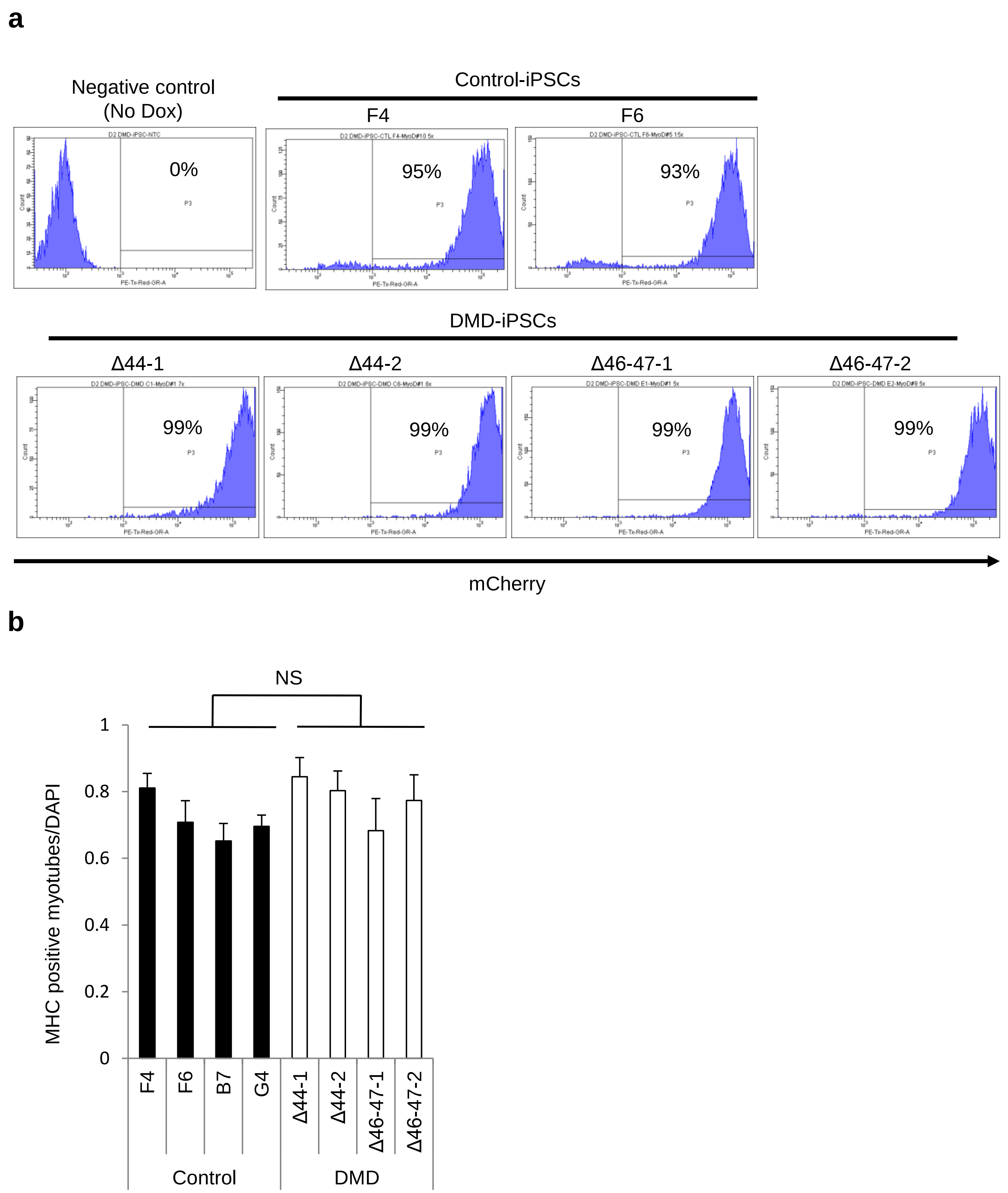

Supplementary Figure S2

(a) Induction efficiency of tetracycline-mediated mCherry expression in hiPSC clones at day 2 differentiation. (b) Differentiation efficiency were calculated based on MHC positive myotubes/No. of nuclei. Two-way ANOVA demonstrated no significant difference between control and DMD groups based on Scheffe’s test.

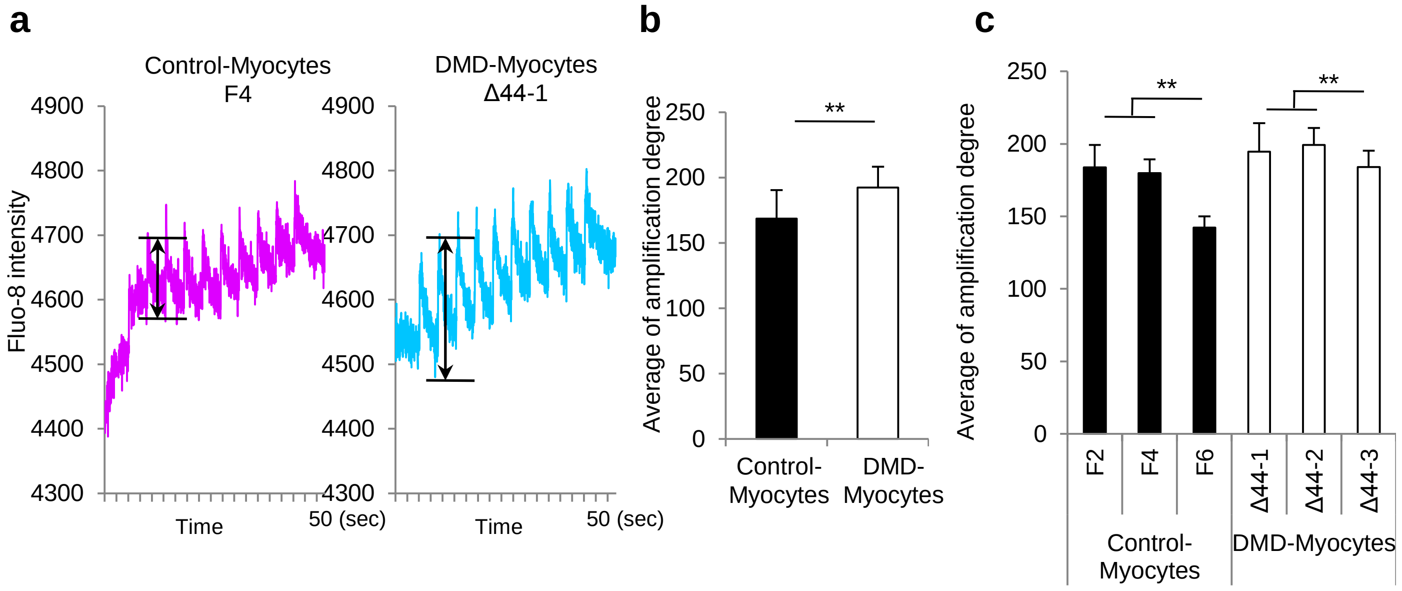

Supplementary Figure S3

(a) Representative profile of Ca^{2+} influx pattern through Fluo-8 intensity in response to electric stimulation. Electric pulse was applied to cells after 5 sec of stationary phase at a constant frequency of 0.2 Hz at 12 V for one minute. The amplitudes are indicated with double arrowheads. (b) Quantitative analysis of Fluo-8 intensity amplitudes in both Control-Myocytes father and Δ44 DMD-Myocytes. n = 12, **$p < 0.01$. (c) Quantitative analysis of Fluo-8 intensity amplitudes in three clones of Control-Myocytes father and DMD-Myocytes Δ44. n = 8, **$p < 0.01$.

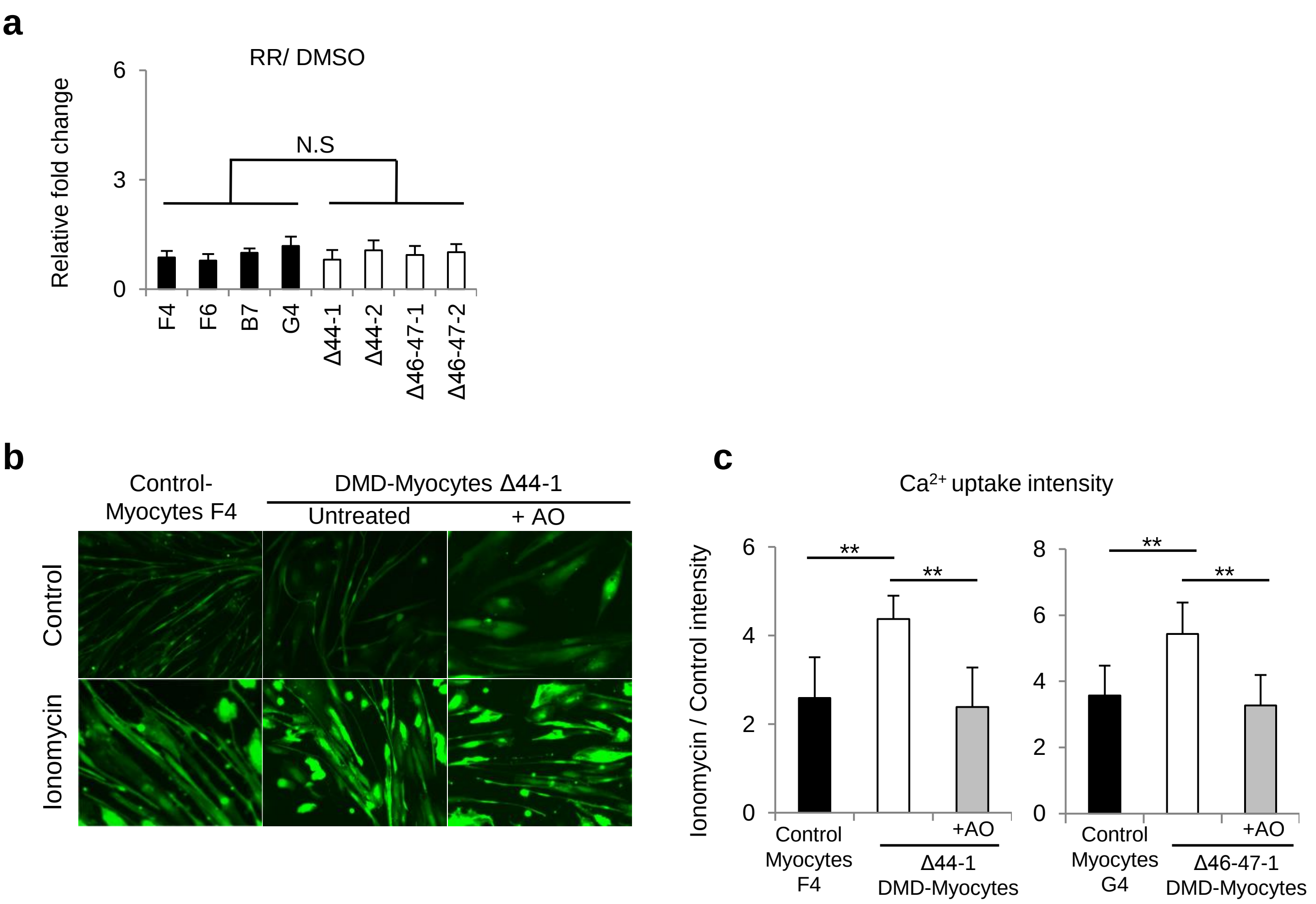

Supplementary Figure S4

(a) CK activity was measured after the addition of RR, TRP family channel inhibitor. CK values were normalised with CK value of DMSO treated samples. (b) Images of myotubes with fluorescent Fluo-8 intensity, visualising Ca^{2+} influx. Scale bar, 200 µm. (c) Ca^{2+} uptake was measured by Fluo-8 intensity in myotubes and quantified. Six myotubes were selected from each Control-, DMD-, and DMD-Myocytes+AO, skeletal muscle cells. Relative fluorescence intensities were normalized to the control intensity in each sample. **P < 0.01 Triplicate experiments for each condition. n = 3.

1st Antibody	Source	Clonarity	Dilution	Company
NCL-DYS1	Mouse	Monoclonal	1/10 (WB)-1/50 (IF)	Leica
NCL-DYS2	Mouse	Monoclonal	1/10	Leica
Dystrophin ab15277	Rabbit	Polyclonal	1/200	Abcam
MHC	Mouse	Monoclonal	1/200	R & D
Skeletal muscle actin	Mouse IgM	Monoclonal	1/200	Acris
CKM	Rabbit	Polyclonal	1/100	Bioworld Technology
TRPV2 (VRL-1)	Rabbit	Polyclonal	1/100	Santa cruz
SSEA4	Mouse	Monoclonal	1/100	Millipore
TRA-1 60	Mouse	Monoclonal	1/100	Millipore
Anti-human nuclei	Mouse	Monoclonal	1/200	Millipore

2nd Antibody	Dilution	Company
Alexa Fluor 488 conjugated goat-anti-mouse IgG	1/500	invitrogen
Alexa Fluor 488 conjugated goat-anti-rabbit IgG	1/500	invitrogen
Alexa Fluor 488 conjugated goat-anti-mouse IgM	1/500	invitrogen
HRP conjugated goat-anti-mouse IgG	1/2000	Vector
HRP conjugated goat-anti-rabbit IgG	1/2000	Vector

Supplementary Table S1 Primary and secondary antibodies used in this study

	Name		Sequence	Annealing temperature	Cycle	RT
RT-PCR	b-actin	Fw	CTCTTCCAGCCTTCCTTCCT	60 ˚C	25	Oligo dT
		Rv	CACCTTCACCGTTCCAGTTT			
	Dystrophin 43-46	Fw	ACAAAGCTCAGGTCGGATTG		35	
		Rv	AGTTGCTGCTCTTTTCCAGGT			
	Dystrophin 43-48	Fw	ACAAAGCTCAGGTCGGATTG			
		Rv	TCCTTCTTGGTTTGGTTGGT			
	Oct3/4	Fw	GACAGGGGGAGGGGAGGAGCTAGG		30	
		Rv	CTTCCCTCCAACCAGTTGCCCCAAAC			
	Nanog	Fw	CAGCCCCGATTCTTCCACCAGTCCC			
		Rv	CGGAAGATTCCCAGTCGGGTTCACC			
	Sox2	Fw	GGGAAATGGGAGGGGTGCAAAAGAGG			
		Rv	TTGCGTGAGTGTGGATGGGATTGGTG			
RT-qPCR	b-actin	Fw	CTCTTCCAGCCTTCCTTCC	60 ˚C	55	
		Rv	CACCTTCACCGTTCCAGTTT			
	Oct 3/4	Fw	GACAGGGGGAGGGGAGGAGCTAGG			
		Rv	CTTCCCTCCAACCAGTTGCCCCAAAC			
	Sox2	Fw	GGGAAATGGGAGGGGTGCAAAAGAGG			
		Rv	TTGCGTGAGTGTGGATGGGATTGGTG			
	Nanog	Fw	CAGCCCCGATTCTTCCACCAGTCCC			
		Rv	CGGAAGATTCCCAGTCGGGTTCACC			
	Exo-MyoD	Fw	ACGTGAGGACGAGCATGTG			
		Rv	GTGCAGCGCTTGAGTGTCT			
	Endo-MyoD	Fw	CACTCCGGTCCCAAATGTAG			
		Rv	TTCCCTGTAGCACCACACAC			
	Dystrophin	Fw	GATGCACGAATGGATGACAC			
		Rv	TGTGCTACAGGTGGAGCTTG			
	Myogenin	Fw	TGGGCGTGTAAGGTGTGTAA			
		Rv	CGATGTACTGGATGGCACTG			
	CKM	Fw	ACATGGCCAAGGTACTGACC			
		Rv	TGATGGGGTCAAAGAGTTCC			
	TPM2	Fw	ACGTGAGGACGAGCATGTG			
		Rv	GTGCAGCGCTTGAGTGTCT			
	Ubiquitin C	Fw	GTAGTCCCTTCTCGGCGATT			
		RV	TTGTCAAGTGACGATCACAGC			

Supplementary Table S2 Primers for RT- and qRT-PCR analysis

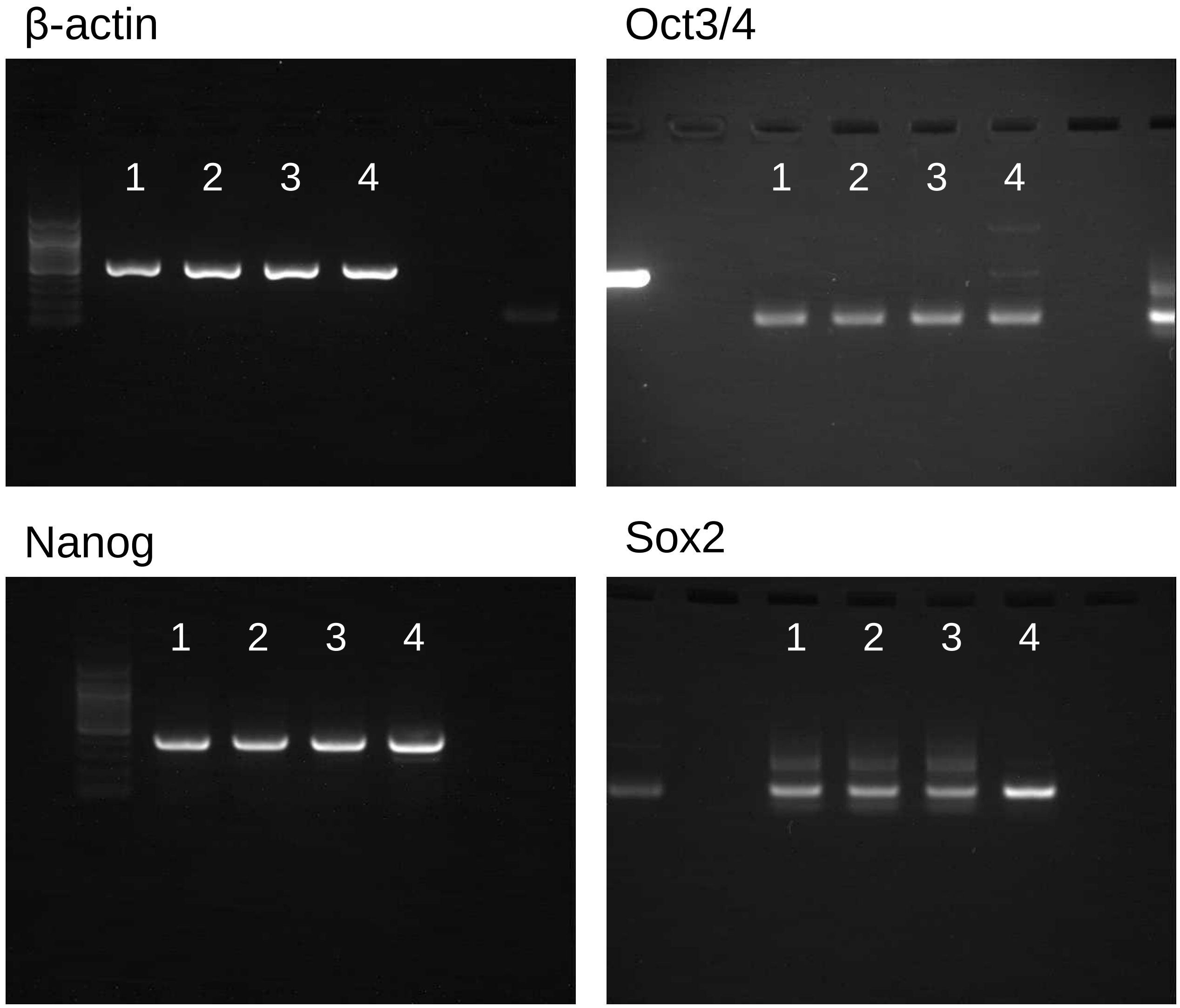

Lane No.
Ladder (100bp DNA)
1: B7
2: Father
3: Δ44
4: Δ46-47

Supplementary Figure Raw data of Figure 1b

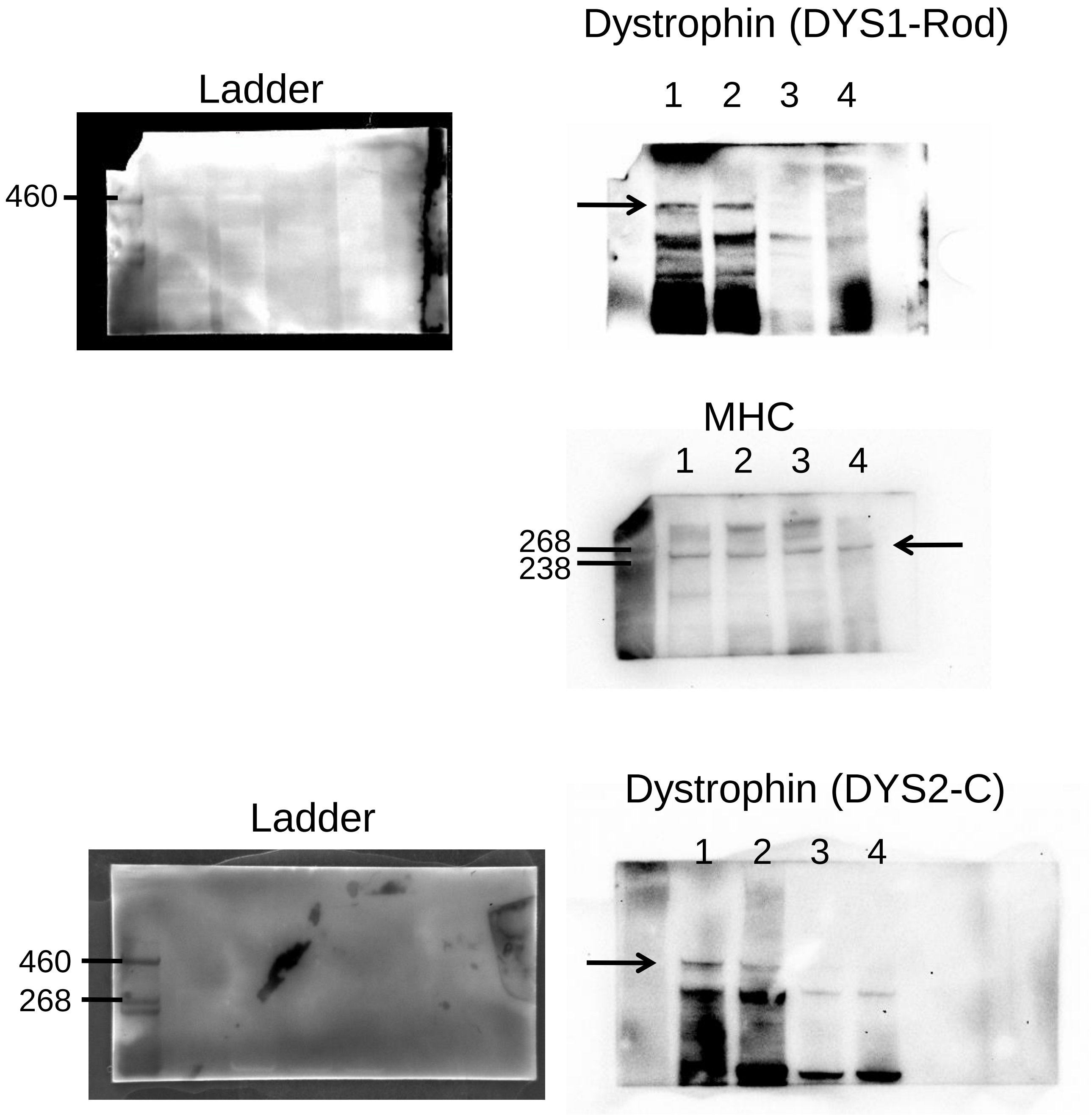

Lane No.
1: B7
2: Father
3: Δ44
4: Δ46-47

Supplementary Figure Raw data of Figure 2d

Δ44

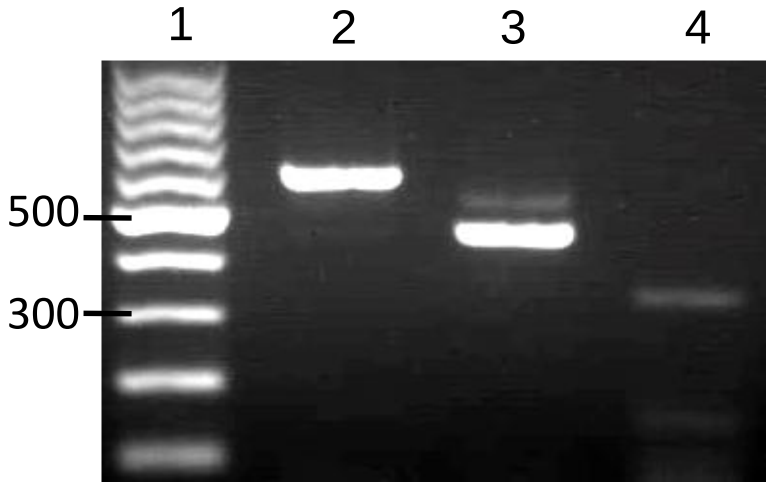

Δ46-47

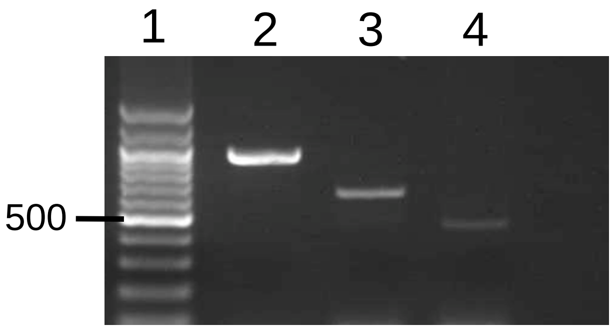

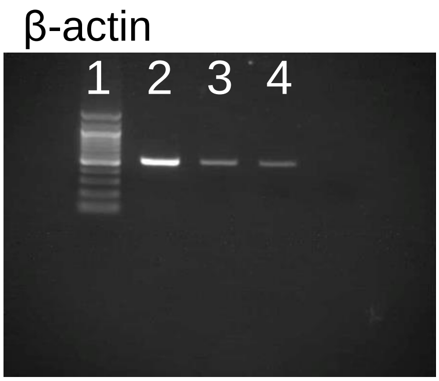

Lane No.
1: Ladder (100bp DNA)
2: Control
3: DMD
4: +AO

Supplementary Figure Raw data of Figure 3b

Δ44

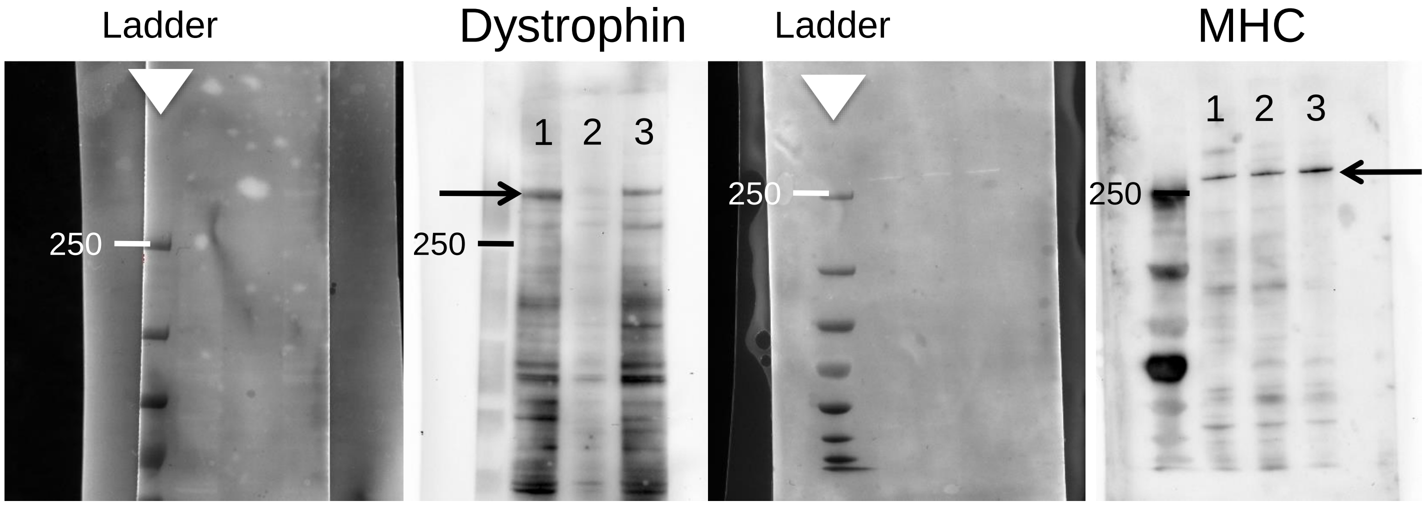

Lane No.
1: Father
2: Δ44-1
3: Δ44-1 +AO

Δ46-47

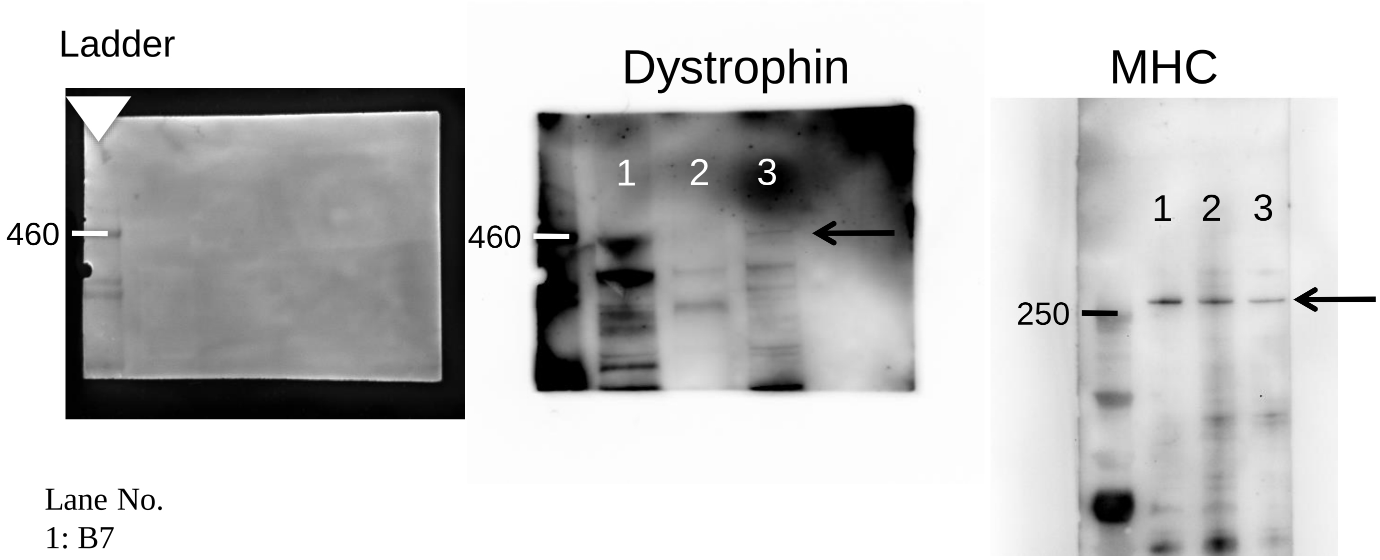

Lane No.
1: B7
2: Δ46-47-1
3: Δ46-47-1+AO

Supplementary Figure Raw data of Figure 3c

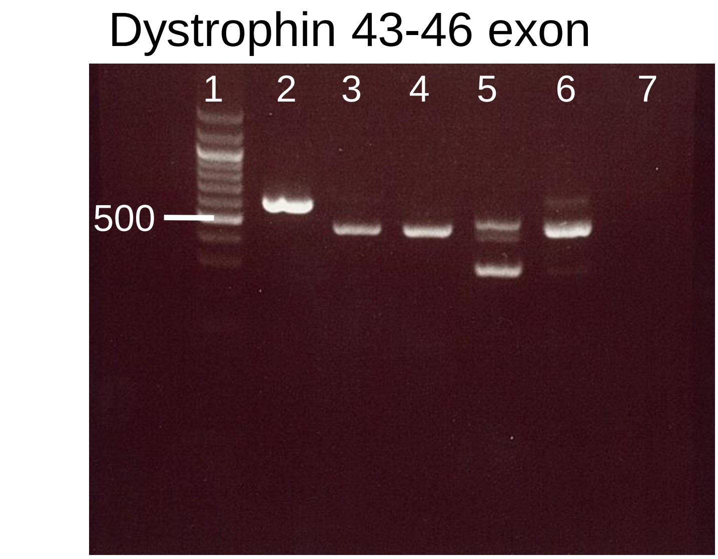

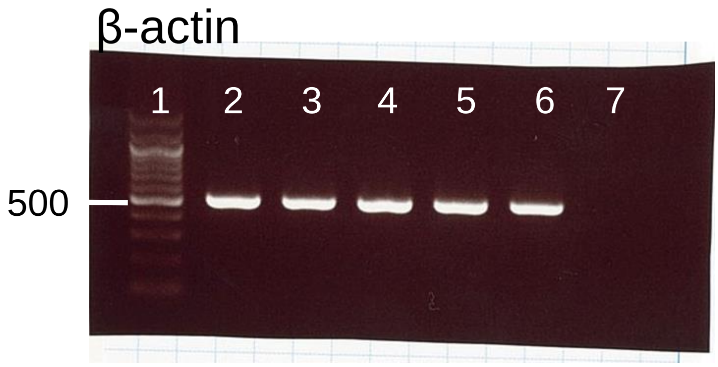

Lane No.
1: Ladder (100bp DNA)
2: Father
3: Δ44-1
4: Δ44-1+CO
5: Δ44-1+AO 200pmol
6: Δ44-1+AO 100pmol
7: Negative control

Supplementary Figure Raw data of Figure 5e
